# Recurrent Headaches and Moyamoya Syndrome in a Non-Asian Descendant: A Case Report

**DOI:** 10.7759/cureus.45748

**Published:** 2023-09-22

**Authors:** Arkaja Singh, Simran Patel, Ayushi Dudhat, Japneet K Bhangu, Dhrumil Patil

**Affiliations:** 1 Internal Medicine, Mahatma Gandhi Medical College and Hospital, Jaipur, IND; 2 Internal Medicine, Government Medical College, Surat, IND; 3 Internal Medicine, California Institute of Behavioral Neurosciences & Psychology, Fairfield, USA; 4 Cardiovascular Medicine, Beth Israel Deaconess Medical Center, Harvard University, Boston, USA

**Keywords:** moyamoya syndrome, moyamoya, cerebrovascular diseases, stroke, migraine, moyamoya etiology, moyamoya disease (mmd)

## Abstract

Moyamoya disease (MMD) is a rare yet progressive cerebrovascular disorder caused by the constriction of arteries, which leads to the twisting and tangling of small arteries in the brain, ultimately causing blockages. Although moyamoya angiopathy (MMA) has been known for almost six decades, its pathophysiology remains unknown, posing challenges to timely diagnosis. Moyamoya syndrome (MMS) refers to the association of MMA with various diseases, including infections, tumors, arteriovenous malformations, radiation treatment, and hereditary disorders. On the other hand, MMD, an idiopathic form, is now more frequently linked to genetic abnormalities. MMS is more common in people of Asian descent, but we encountered and aim to discuss a rare case of a 32-year-old Caucasian from Colombia who was diagnosed with it. The patient initially presented with unexplained symptoms of stroke, prompting doctors to conduct additional imaging. Fortunately, this led to her timely diagnosis. The report discusses the challenges that healthcare professionals face in diagnosis when presented with such uncommon cases. Through this case report, we try to review the presentation, diagnosis, and treatment used for this patient with MMS. The limited information available about the disease, especially the demographic data in countries outside Asia, often leads to delayed diagnoses, emphasizing the need for further exploration. Timelier diagnosis and heightened research into the disease's presentation and risk factors could lead to improved outcomes. Our report also briefly discusses the effectiveness of the current treatment protocol for patients. Currently, the patient is undergoing rehabilitation and showing promising progress.

## Introduction

Moyamoya disease (MMD) is a rare cause of stroke in East Asian countries because of their different genetic makeup as compared to other descents [1]. It is believed that in patients from East Asia with a predisposition for the gene RNF213, which encodes for ubiquitin ligase, the terminal portion of the internal carotid artery (ICA) progressively can get stenosed, which may cause ischemic stroke in these patients [2]. Moyamoya, a type of cerebral vasculopathy, can be either primary (known as MMD) or secondary, often linked to factors such as radiation exposure, sickle cell disease, Down's syndrome, atherosclerosis, neurofibromatosis, hyperthyroidism, bacterial meningitis, fibromuscular dysplasia, Sjögren's syndrome, and certain habits like smoking, oral contraceptive pill use, and cocaine abuse. These factors are believed to contribute to moyamoya syndrome (MMS) development in affected individuals. In contrast, some individuals with a genetic predisposition can develop MMD even without any of these risk factors [2,3]. The distal ICA, including its bifurcation, is the anatomical site of involvement that unites MMD and MMS [4]. MMD is an inherent primary disorder characterized by the gradual narrowing of the anterior intracranial circulation on both sides. This narrowing primarily affects the proximal segments of the intracranial ICA and extends to involve the proximal portions of the anterior cerebral artery (ACA) and middle cerebral artery (MCA). Involvement of the posterior circulation is rare in this condition [2]. In response to this narrowing, a compensatory mechanism is triggered. Numerous smaller vessels, like the lenticulostriate arteries, undergo enlargement and proliferation. This phenomenon is visually depicted in angiograms as a distinctive appearance resembling a "Puff of Smoke," which is translated into the Japanese term "Moyamoya" [2].

The disease is more common in people from East Asia and Caucasians carry a less common non-Arg4810Lys variant of RNF213, which explains the lower prevalence of the disease in European countries and the USA [5]. The data collected for patients outside the Asian population is limited [6], making this case important given her unexpected presentation and absence of common risk factors. This illness results in various combinations of hemorrhagic and ischemic stroke, seizures, and transient focal neurologic deficits. Unilateral MMD is a less common subtype of MMD, accounting for approximately 10-15% of all cases [3]. Bilateral MMD is the more common subtype, affecting both brain hemispheres [7]. In this patient, stenosis at MCA was found without any collaterals, and all the other arteries and circulation were normal.

Moyamoya is associated with headaches, transient ischemic attack, hemorrhagic stroke, seizures, and cognitive impairment [1]. Symptoms of nausea and referred pain in migraine may be due to the involvement of cerebral artery nociceptor as a cause of headache in a patient with MMD [4,8,9]. Headache in MMD is common, and in 63% of patients, headache persists even after successful revascularization [4,10]. This makes us wonder if the treatment used is right or not.

## Case presentation

We present a case involving a 32-year-old Caucasian female from Colombia, South America, with a history of migraine, as well as a family history of stroke in her mother and grandmother, both of whom experienced such events after the age of 60 years. She recently immigrated to the USA with her husband. She sought medical attention at the neurology department due to experiencing complete left-side weakness and numbness over the past three days, with her symptoms progressively deteriorating. Preceding her latest episode, the patient reported nausea, jaw clenching, and the perception of bright spots in her vision. Her husband also observed a concerning incident five days before her most recent presentation: she became unresponsive for 10 minutes while brushing her teeth, followed by noticeable left-sided facial drooping. On the morning of her latest visit to the emergency department, her husband noted changes in her responsiveness and attention, prompting him to seek medical attention again. Her physical examination did not reveal neurological deficits, an inconclusive non-contrast CT scan was obtained, and she was sent home with pain medication for migraine headaches. However, shortly after being discharged, the patient experienced a loss of movement on the whole left side of the body, which made her husband apprehensive, and he sought a second opinion from his family doctor, who advised him to see the neurology division.

Her first-degree cousin exhibited similar symptoms and was later diagnosed with MMS. Although no genetic studies were undertaken, it is worth noting that her migraines, which commenced during her teenage years, were often prompted by stress. She managed these episodes with ibuprofen for symptomatic relief. Lately, stressors related to her immigration to the United States and other personal factors led her to use a combination of caffeine and acetaminophen for her headaches. These medications did not alleviate her suffering, and her headaches became increasingly debilitating. The patient's initial presentation occurred six months prior at an external clinic due to recurrent and progressively worsening headaches. At that time, a non-contrast CT scan yielded inconclusive results, leading physicians to attribute her symptoms to stress-induced headaches and they subsequently discharged her. Over the past six months, the patient underwent two inconclusive CT scans, with no significant findings for either the patient or the attending physicians.

**Figure 1:**
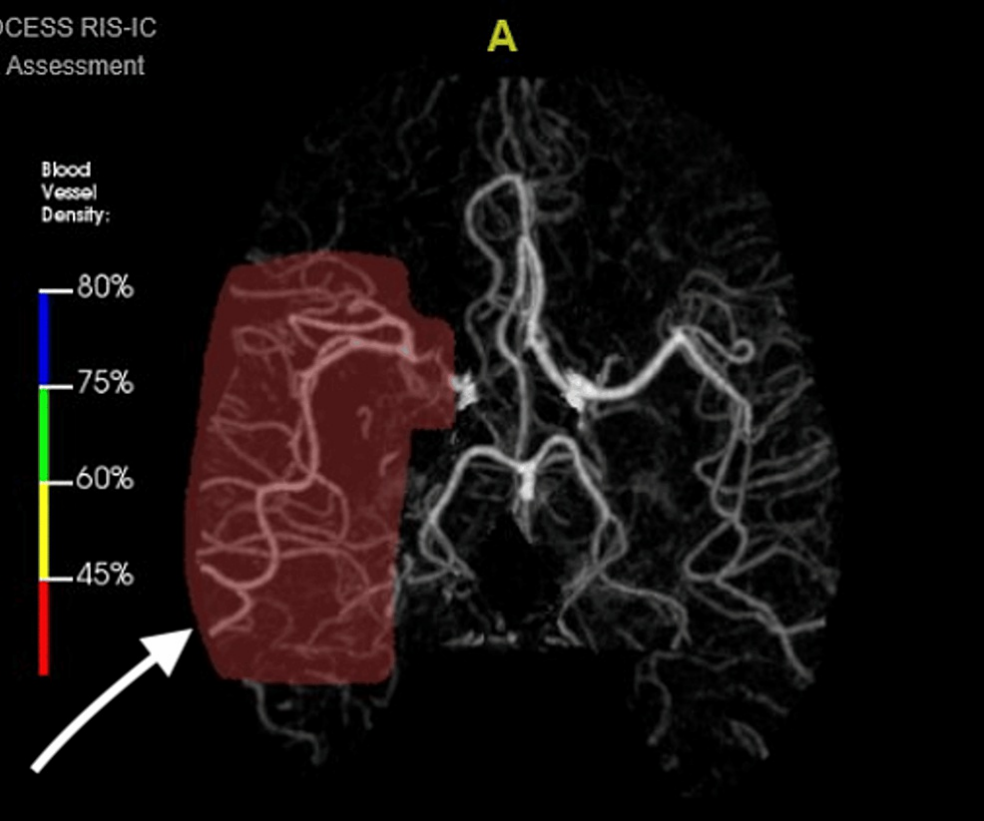
CT angiogram of the brain. Severe stenosis or proximal occlusion of the right A1 anterior cerebral artery segment. Morphology of the occlusive findings suggests chronicity and raises the possibility for vasculopathy such as moyamoya.

**Figure 2:**
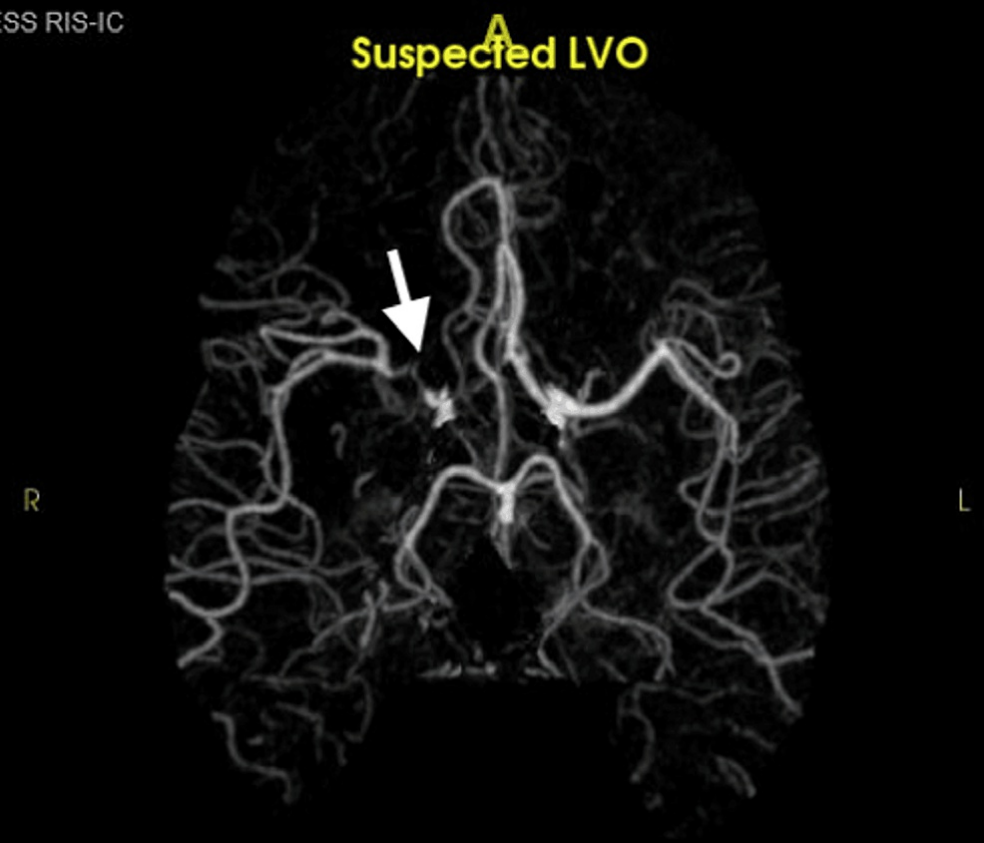
CT angiogram of the brain. A right mid-posterior division M2 middle cerebral artery vessel branch demonstrates high-grade long-segment stenosis. LVO: large vessel occlusion.

**Figure 3:**
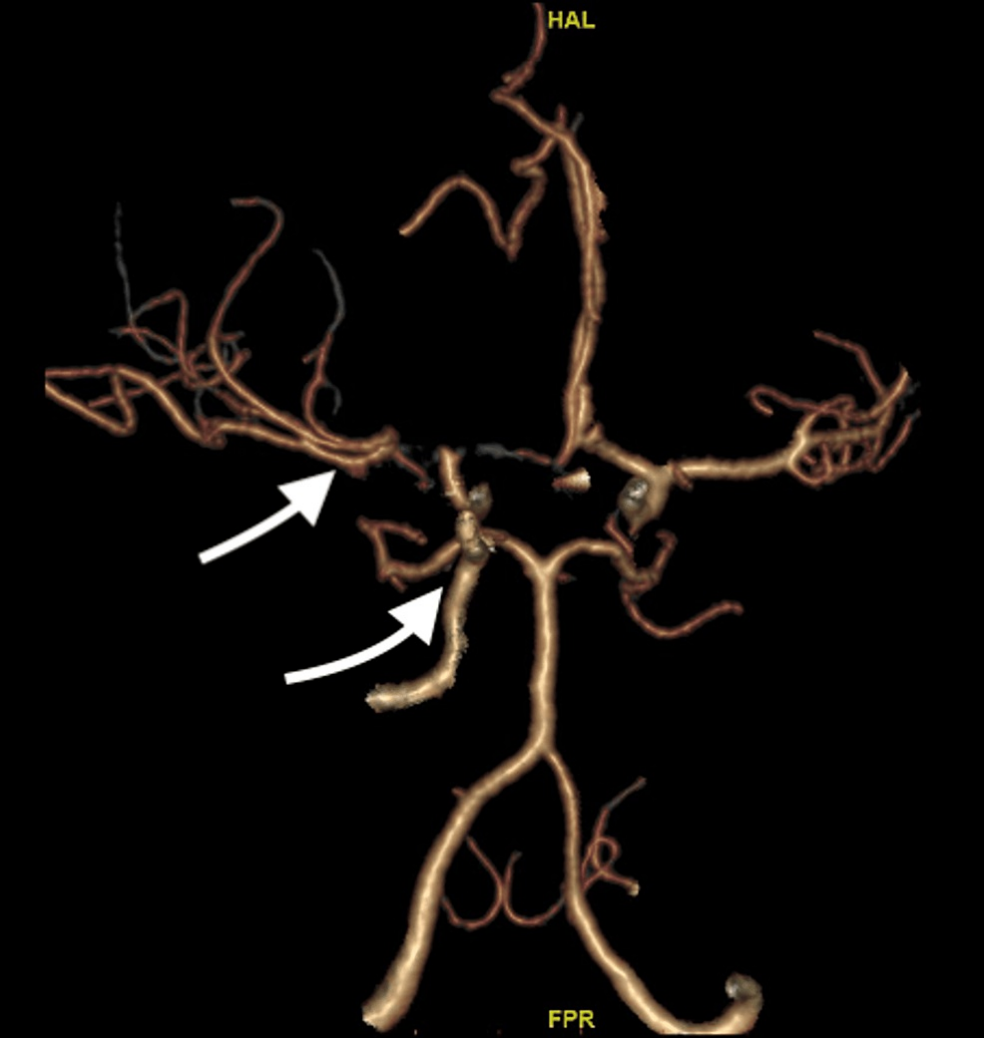
CT angiogram of the head with contrast. Tapered occlusion of the right carotid terminus with non-opacification of the right M1 middle cerebral artery (MCA) (curved arrow). There is distal opacification of the right MCA bifurcation and M2 vessels (straight arrow).

She was admitted to the hospital, and a physical examination revealed intact cranial nerves II through XII. The patient exhibited alertness, responsiveness, and the ability to follow commands. A left facial droop and a reduction in trace finger flexion were noted in the left upper extremity, attributed to a previous infarct in the territory of the right MCA due to unilateral moyamoya vasculopathy (which was also seen on CT angiography imaging (Figure 1) and probably missed on previous CT scan). However, left upper extremity plegia and left lower extremity paresis persisted. There was reduced knee movement, such as flexion and extension, in the left lower extremity and no movement against gravity. CT angiography revealed an occlusion at the terminal end of the right carotid artery and a lack of contrast enhancement in the right M1 segment of the MCA. Additionally, there was a high-grade, extended narrowing observed in a branch of the right mid-posterior division M2 MCA vessel, as depicted in Figure 2. Distal opacification was observed in the M2 vasculature and at the point where the right MCA branches (Figure 3).

**Figure 4:**
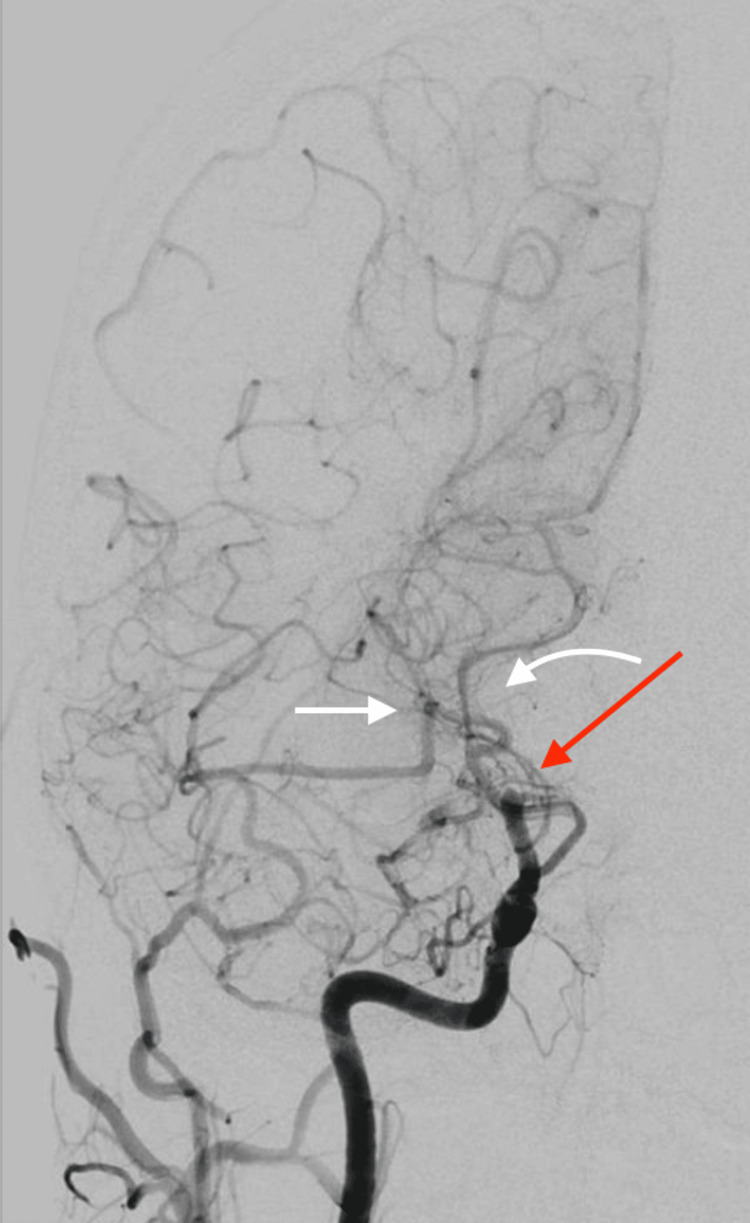
Cerebral angiogram. 1. Moyamoya disease with distal occlusion of the right internal carotid artery (ICA), right M1, and right A1 segments with characteristic moyamoya vessel collateralization within the right middle cerebral artery (MCA) territory filling in a delayed fashion (red arrow). 2. Collateralization from the left anterior circulation to the right anterior circulation through the anterior communicating artery from the right anterior cerebral artery (ACA) to MCA territories, as well as from the posterior circulation through the posterior cerebral artery (PCA) to MCA distal branch vessels (white arrows).

**Figure 5:**
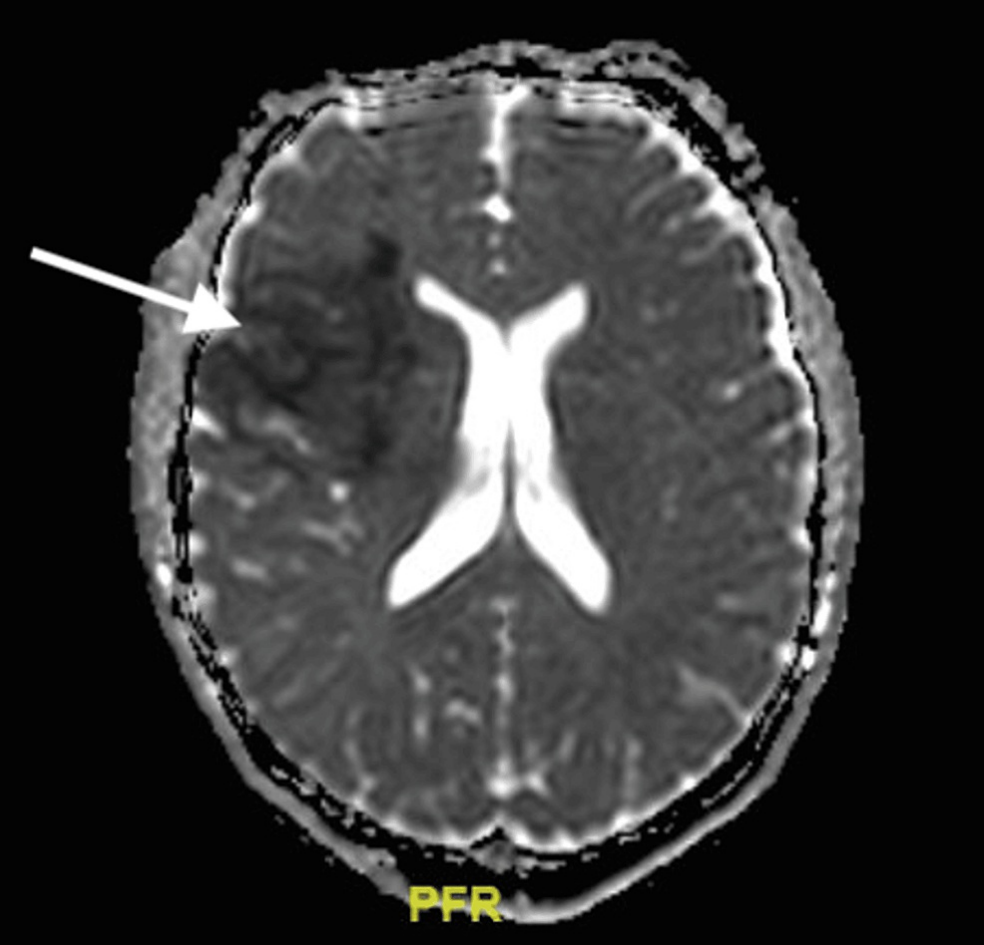
MRI of the brain without contrast. Acute right anterior middle cerebral artery (MCA) infarct involving the right anterior frontal lobe, insula, and right basal ganglia without evidence of significant mass effect or hemorrhage.

The patient underwent a CT angiogram of the head with contrast (Figures 1-3) along with a contrast CT angiogram of cerebral arteries (Figure 4), and an MRI of the brain without contrast (Figure 5). On CT angiography of the neck, there was no evidence of cervical or vertebral artery stenosis. Imaging evidence of moyamoya illness led to the patient's diagnosis of right-sided ischemic stroke.

We also conducted an evaluation to explore potential causes of MMS, including a panel for hypercoagulable states (antinuclear antibody (ANA), antineutrophil cytoplasmic antibody (ANCA), rheumatoid factor (RF), anti-Ro, anti-La, rapid plasma reagin (RPR), and sickle cell screen) along with thyroid tests; however, all these tests returned negative results. Additionally, there was no history of smoking, drug use, Down syndrome, atherosclerosis, or fibromuscular dysplasia.

The patient was admitted to the ICU on the day of presentation to the hospital for close monitoring to reduce the risk of further complications. She was then shifted to the neurology ward the next day for further management. The patient's treatment plan included methylprednisolone, a medication to reduce inflammation. She was started on dual antiplatelet therapy with aspirin (81 mg) and Plavix. To manage pain and headaches, she was given ketorolac injections. Acetaminophen (650 mg) was also provided every six hours to help with headaches. These medications were aimed at addressing her symptoms and providing relief.

The next steps in her care involved recommending bypass surgery to address the narrowing of her ICA. However, before proceeding with the surgery, the plan was for her to undergo thorough rehabilitation therapy to prepare her for the procedure.

She is progressing well and is currently undergoing rehabilitation to address her weakness. There have been notable improvements in her left-side weakness and movements (power ⅗). The patient displayed remarkable improvement: the left lower extremity transitioned from a state of plegia to exhibiting knee movements like flexion and extension, and the patient regained the ability to move her toes, although no movement against gravity. The patient will continue to follow the intensive rehabilitation therapy program, which involves three hours of therapy per day, at least five days per week. She is planned for bypass surgery for the stenosed ICA next month.

## Discussion

Through this case, we observed several difficulties that a physician faces with the presentation that could hinder the diagnosis and treatment of MMS. First, the patient initially experienced headaches and nausea, which were not immediately recognized during the first two assessments. Second, her lack of neurologic deficits prevented the clinicians from investigating it further. A delayed presentation of stroke symptoms could have posed challenges in diagnosing the condition. This case prompts us to include MMD in our list of potential diagnoses when someone presents with similar symptoms when we see a similar case.

MMD is a rare cerebrovascular disorder where the distal ICA and its branches gradually narrow or block. This prompts the growth of compensatory collateral vessels at the base of the brain. Despite significant progress, the exact causes and development timeline of this condition remain unclear. Evidence points to various factors, including angiogenesis, genetics, the immune system, and inflammation. Elements like vascular growth factors and specific cell types have been linked to MMD, along with potential influences such as elastin mRNA, protein expression, and mitochondrial abnormalities [11].

While there is not an extensive body of research on the impact of caffeine, aspirin, and acetaminophen medication on the occurrence of MMD, it is hypothesized that prolonged use of nonsteroidal anti-inflammatory drugs (NSAIDs) could potentially hinder the activity of cyclooxygenase (COX) enzymes, potentially affecting the regular control of cerebral blood flow [12]. It is important to note that using these medications alone is unlikely to cause MMD, as the etiology of the disease is believed to be multifactorial and involves genetic and environmental factors. We may ask if patients with a history of migraine or other cerebrovascular disorders may benefit from avoiding the chronic use of aspirin and NSAIDs. Nevertheless, they should consult with their healthcare provider before taking any medication that may affect cerebral blood flow. The treatment of MMD involves surgical revascularization procedures such as direct or indirect bypass surgery, which aim to improve blood flow to the affected areas of the brain [13]. In cases where surgical intervention is postponed, medical management may be recommended to control symptoms and prevent complications, as in this patient. However, there is not enough information in the published literature at this moment to give reliable recommendations for the appropriate short- and long-term pharmacologic care of these individuals. Further research is the only way we can truly understand the management and approach for patients with MMS.

## Conclusions

In conclusion, MMD is a rare cerebrovascular disorder that can cause significant morbidity and mortality if left untreated. The presence of unilateral involvement is a rare but possible manifestation of the disease. Early recognition and effective management have the potential to prevent severe neurological events, such as strokes, which can have devastating consequences. This case underscores the significance of including MMD as a potential diagnosis when evaluating strokes, especially in younger individuals with a history of recurring headaches. It is crucial not to limit this consideration based solely on the patient's ethnic background. Further research is necessary to establish the relationship between MMD and migraines and to improve our understanding of the pathophysiology of this rare disorder to understand the approach for management.
